# Learning ethical sensitivity through authentic legal experience: an educational psychology perspective on court hearing engagement

**DOI:** 10.3389/fpsyg.2026.1796884

**Published:** 2026-06-12

**Authors:** Xiang Chen

**Affiliations:** College of Science and Technology, Ningbo University, Ningbo, China

**Keywords:** court hearings, ethical intent, ethical sensitivity, experiential learning, perspective-taking

## Abstract

This study examines how engagement with an authentic legal setting may activate ethical sensitivity and prompt reflective moral sense-making in professional education. Drawing on experiential learning and stimulus-based reflection frameworks, it analyses reflective narratives written by undergraduate accounting students following attendance at a court hearing involving professional misconduct. The findings indicate that the court hearing functioned as a temporally distinctive learning context in which moral responsibility was examined but not yet resolved. Ethical sensitivity was initially activated through affective engagement with the courtroom environment and the defendant’s circumstances, prompting reflective sense-making. Empathic perspective-taking emerged as a key psychological mechanism through which empathic engagement supported contextualized ethical judgment, enabling students to balance compassion with professional and legal accountability. In addition, students articulated ethical intent by projecting anticipated responses to future dilemmas, drawing on the court case as a salient moral reference point. Together, the findings highlight how authentic institutional experiences can support learning-related moral development through interconnected affective, cognitive, and future-oriented processes.

## 1 Introduction

Research on ethical learning in professional education has attracted increasing scholarly and public attention, particularly in response to repeated failures of ethical judgment among highly trained practitioners. Such failures raise concerns not only about technical competence, but also about learners’ ethical sensitivity and moral awareness in professional decision-making contexts. Prior studies suggest that individuals involved in serious professional misconduct may possess substantial expertise, yet still display forms of moral blindness that limit their ability to recognize ethical issues embedded in everyday practice (Bazerman and Tenbrunsel, 2011). These concerns have prompted renewed interest in how professional education can help learners recognize moral complexity, interpret responsibility, and reflect on ethical action in situations involving pressure, ambiguity, and institutional constraint.

Conventional approaches to ethics-related instruction have largely relied on classroom-based formats, including lectures, case studies, and hypothetical dilemmas. Although these methods allow learners to analyze ethical issues conceptually, they may offer limited exposure to the emotional, institutional, and human dimensions of professional misconduct (Martinov-Bennie and Mladenovic, 2015). Prior research therefore suggests that purely rule-based or technically oriented approaches may be insufficient to prepare learners for ethically complex professional realities (Loeb and Ostas, 1997; McPhail, 2001; Saat et al., 2010). In response, moral learning research has increasingly emphasized pedagogical approaches that engage learners cognitively, affectively, and reflectively (Dellaportas et al., 2022). Consistent with this emphasis, recent accounting ethics education innovations, including RADAR, Giving Voice to Values, video-based pedagogy, virtual reality learning, and AI-supported role-play, demonstrate a clear shift toward more contextual, reflective, and immersive learning designs (Lee and Massey, 2021; Miller et al., 2020; Ariail and Crumbley, 2024; Ronaghi, 2024; Xiong and Bao, 2025).

Among these innovations, experiential and field-based learning approaches offer a particularly direct way to address the limitations of classroom-based instruction by placing learners in contact with ethically salient situations. Such approaches can elicit emotional responses, disrupt existing assumptions, and prompt deeper reflective processing (Dellaportas and Hassall, 2013; Arcodia et al., 2021). Field-based educational activities, in particular, immerse learners in real-world contexts and have been shown to foster higher-order learning, deepen conceptual understanding, and expose learners to multiple perspectives (Higgins et al., 2012; Kolb and Kolb, 2005; Nadelson and Jordan, 2012). In ethics education, one specialized form of field-based learning is the prison visit, through which students encounter individuals convicted of professional or white-collar crimes. Such experiences can foreground the human, organizational, and institutional consequences of misconduct and encourage empathy, perspective-taking, and more nuanced moral evaluation (Castleberry, 2007; Dellaportas and Hassall, 2013; McPhail, 2003). However, these settings usually involve retrospective reflection after moral responsibility has already been legally and socially resolved.

Less attention has been paid to authentic legal settings in which responsibility is still being examined, interpreted, and publicly adjudicated. From an educational psychology perspective, this is an important gap. Emotionally salient institutional experiences may trigger moral attention, disrupt habitual cognitive frames, and support reflective sense-making, particularly when learners observe ethical responsibility being negotiated in real time. Yet much existing work on ethics education has examined these processes within classroom-based, simulated, or retrospective field-based contexts (Butler et al., 2019). As a result, limited empirical insight is available on how court hearings, as authentic legal-institutional experiences, may activate ethical sensitivity, support reflective ethical judgment, and encourage students to articulate future-oriented ethical intent.

Addressing this gap, the present study examines accounting students’ reflective narratives following attendance at a court hearing involving professional misconduct. Drawing on experiential learning and stimulus-based reflection frameworks, the study investigates how students interpreted a temporally situated legal process in which moral responsibility was publicly examined but not yet fully resolved. In particular, it focuses on how affective engagement, role-based empathic perspective-taking, reflective ethical judgment, and future-oriented ethical intent were articulated in students’ post-hearing reflections. In doing so, this study contributes to educational psychology and professional ethics education by showing how an authentic legal experience may activate ethical sensitivity and prompt reflective moral sense-making.

## 2 Literature review

### 2.1 Ethical sensitivity, ethical judgment, and ethical intent in professional moral learning

Rest’s (1986) four-component model provides a useful framework for distinguishing among ethical sensitivity, ethical judgment, ethical intent, and ethical behavior. In this model, ethical sensitivity refers to the initial recognition that a situation contains morally relevant features. It involves noticing that one’s actions may affect others, identifying who may be harmed or implicated, and recognizing that a professional situation requires moral attention. Ethical sensitivity is therefore a prerequisite for subsequent ethical reasoning, but it is not the same as ethical judgment or ethical intent (Fiolleau and Kaplan, 2017; Martinov-Bennie and Mladenovic, 2015).

Ethical judgment refers to the evaluative process through which individuals assess what is ethically appropriate in a given situation. It is not merely a rule-based assessment, but a context-dependent process shaped by stakeholder consequences, professional responsibilities, and organizational conditions. For example, Onumah et al. (2021) link ethical judgment to the evaluation of alternative actions in relation to stakeholders and the wider public interest, while Musbah et al. (2016) show that ethical judgment is closely associated with ethical recognition and ethical intention. Extending this line of inquiry, accounting and auditing research further identifies multiple influencing factors, including cognitive moral development, client risk, professional values, independence, ethical culture, and perceived organizational pressure (Brandon et al., 2007; Barrainkua and Espinosa-Pike, 2018). In professional education, this implies that students may recognize a situation as morally problematic yet still differ in how they evaluate the seriousness of misconduct, the relevance of contextual pressure, and the scope of professional responsibility.

Ethical intent, by contrast, refers to an individual’s stated willingness or anticipated commitment to act in accordance with an ethical judgment. It is future-oriented and motivational in nature. Ethical intent should also be distinguished from actual ethical behavior, because self-reported intentions do not necessarily demonstrate what individuals will do in practice (Craft, 2012; Yusnaini and Meirawati, 2023). Prior research in accounting ethics suggests that ethical intent is shaped not only by moral evaluation, but also by professional commitment, anticipatory socialization, ethical orientation, attitudes, and organizational context (Buchan, 2005; Elias, 2006; Greenfield et al., 2008). These studies indicate that ethical intent is not simply a personal preference to behave ethically, but a motivational orientation shaped by how individuals connect ethical evaluation with professional role expectations and perceived conditions for action.

These distinctions guide how the present study interprets students’ reflective narratives. Ethical sensitivity is understood as students’ recognition of morally salient features in the court hearing, such as harm, responsibility, legal accountability, and the consequences of professional misconduct. Ethical judgment refers to their evaluative reflections on responsibility, contextual pressure, compassion, and professional accountability. Ethical intent refers to their stated future-oriented responses to similar ethical challenges. In this way, the study treats ethical sensitivity, ethical judgment, and ethical intent as related but conceptually distinct aspects of students’ reflective moral sense-making. Consistent with this cautious stance, the study does not claim to demonstrate sustained ethical development or actual behavioral change, but rather examines how an authentic legal experience appeared to activate these processes in students’ immediate reflections.

### 2.2 Experiential learning, reflection, and learning-related moral development

Research on moral learning suggests that ethical understanding is unlikely to develop through the transmission of ethical rules or abstract principles alone (Dewey, 1938; McPhail, 2001, 2003). Instead, learners recognize and interpret ethical issues through engagement with lived experience, particularly experiences that involve moral complexity, emotional tension, and competing values (Dewey, 1938; McPhail, 2003). From an educational psychology perspective, moral learning is therefore not only a cognitive process, but also an experiential and reflective one.

Experiential learning theory provides a useful framework for explaining this process, emphasizing the transformation of experience through reflection and interpretation rather than the accumulation of knowledge (Kolb, 1984; Kolb and Kolb, 2005). In ethically salient contexts, experience may elicit discomfort, empathy, moral unease, or surprise. These affective responses can direct learners’ attention to morally relevant features of a situation and disrupt habitual or purely rule-based interpretations (Krathwohl et al., 1964; McPhail, 2003). This is particularly important in professional education, where technical routines and institutional pressures may obscure the moral dimensions of decision-making.

Affective engagement is central to the activation of ethical sensitivity. Emotions signal that a situation requires moral interpretation and reflective attention (Lerner and Keltner, 2000; Immordino-Yang and Damasio, 2007). Reflection then allows learners to examine, interpret, and connect these emotional responses to professional values and responsibilities. Through reflective engagement, learners move beyond immediate reactions toward perspective-taking, consequence evaluation, and consideration of role-based obligations (Rest, 1986; Bérubé and Gendron, 2023). In this way, affective activation and reflection jointly support ethical sensitivity and lay the groundwork for subsequent ethical judgment and ethical intent.

Recent accounting education research cautions that reflection should not be treated as an automatic outcome of experience. Dellaportas et al. (2022) argue that reflective practice is central to connecting experience with professional judgment, values, and future action. Wilkin (2022) adds that critical reflection requires deliberate educational support rather than passive exposure, while Daff et al. (2024) show that course design can encourage reflective habits by structuring opportunities to examine experience, judgment, and professional responsibility. For the present study, this suggests that the educational value of a court hearing lies not in physical attendance alone, but in how the experience stimulates reflection on moral responsibility, professional role expectations, and possible future conduct.

From a stimulus-based learning perspective, external events prompt learning when they interact with learners’ existing assumptions and frames of reference (Wilson and Beard, 2003). Experiences that challenge prior beliefs or expose tensions between personal values, professional norms, and real-world consequences are especially likely to stimulate reflective moral sense-making (McPhail, 2003; Dellaportas and Hassall, 2013). In this study, the court hearing is conceptualized as such a learning stimulus. Its educational significance derives from the combination of formal institutional authority, public examination of professional misconduct, and emotionally salient exposure to the defendant’s circumstances and the consequences of wrongdoing.

This framing clarifies the explanatory pathway examined in the study. The court hearing may first activate moral attention through emotional responses (e.g., discomfort, empathy, tension, surprise). These responses then prompt reflection, as students reconsider initial assumptions and engage in role-based empathic perspective-taking. Through this reflective process, students may develop more contextualized ethical judgment by weighing situational pressure, personal hardship, professional responsibility, and legal accountability. Finally, such reflective judgment may support the articulation of future-oriented ethical intent, including anticipated refusal of misconduct, willingness to seek guidance, intention to intervene, or use of the court case as a moral reference point. Consistent with recent work on reflective practice (Dellaportas et al., 2022; Wilkin, 2022; Daff et al., 2024), this sequence underscores that the value of experiential learning depends not only on authentic settings but also on learners’ reflective interpretation of those experiences.

### 2.3 Field-based moral learning environments

Within the growing literature on experiential and reflective pedagogies in accounting education (Gittings et al., 2020; Dellaportas et al., 2022; Van Akkeren and Tarr, 2021; Miller and Willows, 2024), field-based learning environments are especially relevant because they situate learners within authentic social and institutional settings where ethically salient issues are encountered as part of practice rather than as abstract classroom problems. Prior research suggests that exposure to real-world contexts can support ethical sensitivity by embedding moral judgment within concrete relationships, institutional norms, and tangible consequences (Dewey, 1938; McPhail, 2003; Nadelson and Jordan, 2012). Compared with classroom-based instruction, such environments may elicit affective engagement and perspective-taking, thereby directing attention to moral issues and supporting reflective sense-making.

Within professional education, several experiential and field-based formats have been employed to support ethical learning. Existing reviews suggest that experiential learning activities in accounting education are diverse, but that relatively few directly engage students with economic crime, legal accountability, or institutional processes of responsibility assignment (Gittings et al., 2020). One well-documented approach involves prison visits, in which students engage with individuals convicted of professional or white-collar crimes. Such experiences confront learners with the human and social consequences of unethical behavior and have been shown to foster empathic engagement, moral reflection, and more nuanced understanding of ethical failure (Castleberry, 2007; Dellaportas and Hassall, 2013). Another strand of research has examined simulated legal environments, particularly mock trials, which engage learners in role-taking, legal reasoning, and structured reflection within a pedagogically designed setting (Van Akkeren and Tarr, 2021). Together, these studies demonstrate that experiential formats can support ethical learning beyond rule-based instruction by engaging affective and cognitive dimensions of learning.

Despite their pedagogical value, existing field-based interventions capture only a limited range of psychologically salient moral experiences. Prison visits typically situate ethical learning after ethical failure has been judged and sanctioned, thereby emphasizing retrospective moral evaluation (McPhail, 2003; Dellaportas and Hassall, 2013). Mock trials, while enabling active role-taking and guided reflection, rely on designed scenarios and pedagogical scripting that attenuate institutional authority and real-time accountability (Van Akkeren and Tarr, 2021). As a result, both approaches may constrain learners’ exposure to the kinds of emotionally and institutionally situated stimuli through which ethical sensitivity is activated in authentic professional contexts.

Taken together, this literature suggests that accounting ethics education has moved toward more experiential, reflective, and context-rich approaches, including classroom-based ethical reasoning models, role-play, virtual learning, mock trials, prison visits, internships, and field-based activities (Miller et al., 2020; Poje and Zaman Groff, 2022; Dellaportas et al., 2022; Van Akkeren and Tarr, 2021; Ronaghi, 2024; Xiong and Bao, 2025). Yet authentic court hearings remain comparatively under-examined as learning environments. Unlike simulations or mock trials, court hearings expose learners to unscripted institutional authority, procedural formality, and real consequences. Unlike prison visits, they allow students to observe responsibility as it is being examined and adjudicated rather than only after punishment has occurred. By examining accounting students’ reflective responses to attending a court hearing, the present study addresses this gap and advances understanding of how authentic legal exposure may activate ethical sensitivity, support reflective ethical judgment, and encourage students to articulate future-oriented ethical intent.

## 3 Research method

### 3.1 Research context and participants

Participants were third-year undergraduate accounting students enrolled in a four-year accounting program at a university in northern China. The program includes ethics-related content and places emphasis on professional responsibility and ethical reflection. Ethical approval for the study was obtained from the university’s Research Ethics Committee prior to data collection.

All third-year undergraduate accounting students in the relevant cohort were invited to attend the court hearing and were informed of its educational purpose. Due to courtroom seating constraints, 100 students were randomly selected from those who expressed interest. Of these, 95 were able to participate on the day of the hearing and completed the reflective task; the remaining five did not attend for personal reasons, such as scheduling conflicts.

Table 1 reports the demographic and academic characteristics of the 95 students who attended the court hearing and completed the reflective questionnaire. The participants were all third-year accounting students, with a mean age of 21.6 years. The group was predominantly female, broadly reflecting the gender composition of the wider interested cohort. Participants also varied in education level, academic major, prior ethics course enrolment, and prior work experience. These characteristics suggest that the final reflective sample broadly represented the wider pool of students who had expressed interest in attending the court hearing.

**Table 1:** Participant characteristics of students who attended the court hearing.

Variable	Category	Participants (*n* = 95)
Year of study	Third year	95 (100%)
Gender	Female	64 (67.4%)
	Male	31 (32.6%)
Age	Mean (SD)	21.6 (0.7%)
Education level	Bachelor’s program	66 (69.5%)
	Junior college	29 (30.5%)
Academic major	Financial accounting	46 (48.4%)
	Auditing	49 (51.6%)
Prior ethics course enrolment	Enrolled	45 (47.4%)
Prior work experience	None	45 (47.4%)
	Has experience	50 (52.6%)

### 3.2 Reflective data collection

Data were collected using an open-ended reflective questionnaire designed to capture students’ moral reflections following their attendance at the court hearing. Consistent with interpretive traditions in educational psychology and moral learning research, the instrument focused on how students perceived, interpreted, and made sense of ethically salient aspects of the experience, rather than measuring ethical sensitivity as a stable individual trait.

The questionnaire was adapted from Dellaportas and Hassall (2013), who examined experiential ethics education through students’ reflective accounts of a prison visit. It comprised four open-ended questions broadly informed by experiential learning theory (Kolb, 1984), inviting participants to describe what they observed during the court hearing, how they interpreted the experience, what ethical meanings they derived from it, and how it might influence their future professional conduct. This structure enabled students to articulate affective, cognitive, and behavioral reflections in their own words.

All questions were deliberately open-ended to encourage depth of reflection rather than predetermined responses. The questionnaire was piloted with a small group of accounting students who did not attend the court hearing, and minor revisions were made to improve clarity and ensure that the questions elicited reflective rather than purely descriptive responses. The full set of questions is presented in Table 2.

**Table 2:** Reflective prompts and conceptual influences.

Experiential orientation	Survey question
Concrete experience (CE)	What happened during the court hearing? What were your thoughts, feelings, and perceptions?
Reflective observation (RO)	What did you understand from the court hearing experience?
Abstract conceptualisation (AC)	What have you learned from this experience?
Active experimentation (AE)	What are your future behavioral plans? Would this experience influence how you make decisions in similar situations?

Source: Kolb (1984).

Students completed the reflective questionnaire in Chinese. The quotations presented in the findings were translated into English by the authors. The translations were meaning-based rather than strictly literal, with minor editing for grammar and readability. Care was taken to preserve the original meaning, tone, and substantive content of students’ responses. No quotations were paraphrased beyond what was necessary for clear English presentation. The translations were also reviewed twice by a departmental colleague who was not involved in the data collection to verify semantic consistency, and no major discrepancies were identified.

### 3.3 Reflexivity and ethical safeguards

Because the participants were students in an educational setting, the study involved a potential power relationship between students, instructors, and the researcher. Several safeguards were therefore adopted to reduce the influence of this relationship on students’ participation and responses. The reflective task was used for research purposes only. It was not part of formal coursework, was not assessed, and did not contribute to students’ course marks or academic standing. Students were informed that participation was voluntary and that they could decline to complete the reflective questionnaire without penalty. They were also assured that the content of their responses would not be shared with course instructors in an identifiable form and would not affect their academic evaluation.

To further reduce social desirability pressure, responses were collected anonymously, and students were invited to describe their own perceptions and reflections rather than provide a “correct” ethical answer. The course instructor was not involved in collecting the questionnaires or reading identifiable responses.

### 3.4 Data analysis

The open-ended responses were analyzed using thematic analysis informed by an interpretive qualitative approach. The analysis proceeded in several stages.

First, all 95 responses were read repeatedly to develop familiarity with the dataset. During this stage, initial codes were generated inductively from segments of text that reflected students’ emotional reactions, recognition of moral issues, interpretations of responsibility, empathic responses, references to professional obligation, and stated future intentions. The coding remained close to participants’ own language in order to preserve the meaning of their reflections.

Second, related codes were compared and grouped into broader thematic categories through an iterative process of constant comparison. Codes expressing similar meanings were combined, while ambiguous or overlapping codes were revisited in relation to the original responses. For example, codes relating to emotional discomfort, sympathy, and moral unease informed the theme of affective moral awareness; codes relating to perspective-taking, contextual pressure, and professional accountability informed the theme of reflective ethical judgment; and codes relating to refusal of misconduct, help-seeking, intervention, and recalling legal consequences informed the theme of future-oriented ethical intent.

Third, the emerging themes were then reviewed and refined to ensure internal coherence and conceptual clarity. Initial coding was developed inductively from students’ responses and stayed close to their original language. In later stages, the analysis drew on ethical sensitivity, ethical judgment, and ethical intent as guiding concepts, without using them as fixed coding categories. The final themes were developed through repeated comparison between the data, the initial codes, broader categories, and relevant theoretical ideas.

To strengthen the credibility of the interpretive analysis and reduce the influence of single-rater bias, the initial coding and theme development were led by the first author. A research colleague who was not involved in the data collection independently reviewed the coding structure, theme definitions, and the linkage between initial codes, final themes, and the study’s guiding conceptual constructs. This review considered whether the themes were adequately supported by the student responses, whether theme boundaries were sufficiently clear, and whether the interpretation remained grounded in the data rather than primarily driven by the theoretical framework. Where ambiguities or overlaps were identified, the relevant excerpts were re-examined against the original student responses, and theme boundaries were refined accordingly.

Analytic memos were maintained throughout the analysis to record coding decisions, emerging interpretations, revisions to theme definitions, and reflections on possible researcher influence. These memos, together with the coding structure presented in Table 3 and the summary of participant codes in Appendix, formed an audit trail that supported the transparency and traceability of the analytical process, thereby helping to ensure that the final themes were developed through sustained engagement with the data and reflexive consideration of the researcher’s interpretive role.

**Table 3:** Coding structure, analytical themes, and theoretical interpretation.

Stage of moral sense-making	Analytical theme	Core features of students’ reflections	Dimension of ethical sensitivity
Affective moral awareness	Emotional activation through court exposure	Initial emotional reactions to the defendant and courtroom setting; experiences of moral discomfort, empathy, and emotional tension; disruption of initial rule-based judgments	Recognition of moral issues; emotional awareness of ethical salience
Empathic and reflective moral judgment	Contextualized evaluation of responsibility	Reassessment of professional responsibility through consideration of intent, pressure, role, and consequences; balancing compassion with accountability; perspective-taking across multiple actors	Ethical judgment; perspective-taking; proportional evaluation of responsibility
Future-oriented ethical intent	Anticipated ethical action and moral agency	Articulation of intended responses to future ethical dilemmas; resistance to misconduct; willingness to intervene or seek support; use of the court case as a moral reference point	Ethical intent; value alignment; commitment to ethical action

To make the analytical process more transparent, Table 3 presents the coding structure, final themes, and theoretical interpretation used in the analysis. It illustrates how recurring patterns in students’ reflections were developed from initial coding focuses into broader analytical categories and then linked to the study’s conceptual distinctions among ethical sensitivity, ethical judgment, and ethical intent. In addition, Appendix provides a summary of participant codes by theme and sub-theme to further illustrate the distribution of supporting responses across the dataset.

### 3.5 Court hearing context

The court hearing observed in this study involved a case of accounting-related fraud in which a professional accountant was charged with misappropriation of company assets. The case was selected in consultation with course instructors to ensure its relevance to students’ disciplinary background and its potential to raise ethically salient issues related to professional responsibility, integrity, and accountability.

The hearing took place in a public court and followed standard judicial procedures. Students observed the formal presentation of evidence, the examination of facts, and the articulation of legal responsibility by judicial actors. The proceedings took place in a formal courtroom setting marked by clear procedures, visible authority, and public scrutiny. These features made issues of professional misconduct, ethical responsibility, and legal accountability visible as the case unfolded, prompting students to attend more closely to the moral dimensions of the situation.

To protect the privacy of individuals involved, no identifying information is disclosed, and pseudonyms are used where necessary. Students attended the hearing as observers and did not interact with court personnel or participants. Following the hearing, students were invited to reflect on their experience through the survey instrument described above.

## 4 Findings

### 4.1 Affective moral awareness

Students’ affective reflections revealed a marked progression in moral awareness as they engaged with the court hearing. Prior to attending the hearing, many students formed strongly negative impressions of the defendant based on the case facts, frequently characterizing her as selfish, greedy, and unethical. These initial responses reflected a rule-based form of moral evaluation, focused primarily on the violation of professional norms and legal obligations. As one student stated, “My first impression of her was not good. I saw her as someone who abused her power, acted recklessly, and disregarded the law. She violated the fundamental professional ethics expected of a financial officer” (#0539, Male).

As the hearing unfolded, students were exposed to the defendant’s personal circumstances and observed her demeanor directly. This exposure generated emotional tension and moral discomfort, as students found it difficult to reconcile their preconceived image of a fraudulent accountant with the defendant’s ordinary appearance and personal narrative. The tension intensified when it emerged that her actions were motivated by the need to pay for her son’s medical treatment. At this point, many students began to reassess their initial judgments. One participant reflected, “I sense that Anna was compelled by her circumstances. The challenges of divorce and raising a sick child alone, combined with economic and psychological pressure, led to overwhelming stress that ultimately drove her to transgress” (#0527, Female).

This shift did not imply a denial of wrongdoing. Rather, students increasingly distinguished between the unethical nature of the act itself and the situational factors influencing the defendant’s behavior. Moral evaluation became more differentiated, with greater attention paid to motivation, pressure, and relative responsibility. Some students began to view the defendant not as a principal instigator driven by greed, but as an accomplice acting under severe constraint. This reframing suggests a shift away from categorical moral condemnation toward more contextualized and proportionate ethical judgment.

Not all students responded to the hearing in the same way. While many expressed empathy toward the defendant, others retained a more accountability-first interpretation, emphasizing that personal hardship could not excuse professional misconduct. These responses indicate that affective engagement did not uniformly lead to increased sympathy or contextual mitigation. For some students, the hearing reinforced a rule-oriented understanding of professional responsibility, in which legal accountability and professional duty remained the primary basis for moral evaluation.

Taken together, these responses suggest that the court hearing served as a salient moral experience for students. Being present in a real courtroom, where legal authority and human consequences were visibly intertwined, prompted emotional engagement and encouraged students to reflect more deeply on the ethical dimensions of the case.

### 4.2 Empathic and reflective moral judgment

Building on the affective shifts described in the preceding theme, students’ reflections revealed a prominent process of empathic engagement rooted in empathic perspective-taking. Rather than responding with detached sympathy, many students described imagining themselves in the defendant’s position and reflecting on how they might act under comparable personal and professional pressures. This process enabled students to move beyond abstract moral evaluation toward a more relational and self-referential form of ethical understanding.

This empathic perspective-taking appeared to operate at two interrelated levels.

The first level involved emotional resonance with Anna’s immediate suffering and perceived sense of loss. One participant reflected on her imagined longing for family connection: “Locked away in prison, she must have yearned for the days when she could spend every moment with her children.” (#0536, male).

Another student explicitly identified with Anna’s role as a mother, stating:

“If I were in Anna’s shoes, my foremost concern would be my child’s well-being. He is still battling illness, requiring medical procedures with significant expenses. If my misconduct were to force me to be separated from him, I would feel an immense sense of regret for my children.” (#1022, female).

Through these emotionally grounded reflections, students moved beyond observation to form more personal connections with the defendant’s circumstances. These responses suggest that students were engaging with the case in a manner that involved both emotional involvement and self-referential reflection.

A second level of empathic perspective-taking centered on students’ consideration of the broader consequences of Anna’s actions for others. Participants frequently referred to the anticipated impact on Anna’s son, describing concerns about his health, family stability, and emotional well-being. One student commented:

“I believe the child is the most unfortunate individual in this situation. He had already experienced the challenges of his parents’ divorce and an unstable family environment. Now, on top of all that, he is battling illness and unable to be with his mother. Furthermore, the family’s financial stability has been jeopardized. The child’s medical expenses, basic necessities, and transportation are all in question. This sense of hopelessness is truly heart-wrenching.” (#0806, female).

Students also recognized the burden placed on Anna’s parents as indirect victims of the misconduct. As one participant noted:

“I think Anna’s parents are the real victims here. Because she’s divorced, right? So, naturally, the whole responsibility of looking after her sick kids just lands straight on her parents’ shoulders. And on top of all that, they have got to deal with all those weird stares and judgments from people around them.” (#7522, female).

In addition, some students employed metaphorical language to articulate the internal conflict they perceived in Anna’s decision-making. One student described the dilemma as a struggle between rational judgment and emotional pressure: “It’s like standing on the edge of a cliff, with reason pulling her toward the sunlit side on the other end of the cliff, while emotions compelled her toward the darkness.” (#0534, female).

Through this empathic engagement, students’ moral judgment also became more reflective and proportionate. Rather than evaluating misconduct solely in terms of rule compliance or legal violation, students increasingly assessed ethical responsibility in relation to professional role, moral agency, and the capacity to intervene under pressure.

Engagement with the court proceedings prompted students to reconsider how professional authority shapes moral responsibility. While acknowledging the involvement of other actors in the fraudulent scheme, many concluded that the accountant bore primary responsibility because her professional knowledge and position enabled her to prevent the misconduct. As one student observed, “If Anna had refused from the outset, declaring such activities illegal, the misconduct would not have occurred” (#0622, Female).

This response represents an accountability-oriented interpretation rather than a sympathy-driven one. It shows that some students’ reflections remained strongly anchored in professional duty and legal responsibility, even when they recognized the pressures surrounding the defendant’s actions.

This judgment reflects a shift from viewing professionals as passive participants in organizational processes toward recognizing their role as moral gatekeepers with an obligation to resist unethical conduct.

Taken together, these reflections show that empathic perspective-taking helped students move from emotional engagement toward more reflective moral judgment. By imagining the personal and relational consequences of misconduct, students reassessed ethical responsibility in relation to professional role, agency, and their capacity to intervene under pressure. Importantly, empathic engagement did not lead to moral leniency, but supported more balanced evaluations in which compassion for personal hardship coexisted with recognition of professional accountability.

### 4.3 Behavioral reflection and ethical intent

Building on the cognitive reframing and empathic engagement described in the preceding themes, a third theme concerned how students articulated intended responses to future professional dilemmas. Rather than expressing abstract commitments to ethical conduct, students described concrete intentions and decision-making strategies for navigating ethically challenging situations, indicating the formation of ethical intent as a forward-looking orientation.

A prominent pattern in students’ responses was a firm refusal to engage in illegal activities, coupled with an expressed willingness to intervene when encountering misconduct by others. Rather than limiting their responses to personal non-involvement, several students described proactive actions aimed at discouraging unethical behavior. One student stated, “I will try to dissuade the individual from pursuing the criminal scheme. If the salesperson persists, I will closely monitor their subsequent actions” (#1129, female). These responses indicate that students considered intervention and communication as part of their anticipated professional responsibilities.

In addition, students frequently described approaching ethical dilemmas as situations requiring active problem-solving rather than passive endurance. Many outlined specific resources they would seek when facing ethical pressure, including organizational channels such as supervisors, as well as personal support networks such as family members or friends. For example, one student explained, “The first person I would think of seeking help from would be my family. At the same time, I will actively confess all the details to my immediate supervisor.” (#0952, male). These reflections suggest that students recognized the availability of multiple avenues for addressing ethical challenges.

However, not all students articulated responsibility-oriented intent. A subset of responses reflected a more deterrence-oriented perspective, focusing on punishment, imprisonment, shame, family disruption, or personal loss as reasons for avoiding misconduct. These accounts suggest that the court hearing sometimes operated less as a source of professional value internalization and more as a vivid warning about the consequences of unethical conduct.

“Every action has its consequences, and as accountants, we face daily temptations involving money. The thought of facing the same fate as Anna, standing in the defendant’s dock, dealing with a shattered family, and enduring imprisonment naturally curbs any inclinations toward greed.” (#1087, male).

Similarly, another student emphasized the enduring impression of legal sanctions:

“Although engaging in illegal activities may seem to bring us good fortune at first, such “luck” will not last. Once exposed, we will face legal sanctions, and by then, the loss will far outweigh any initial gains.” (#0536, female).

Taken together, these accounts show that students’ articulated ethical intent varied in orientation. Some responses reflected responsibility-oriented intent, including refusal of misconduct, help-seeking, and willingness to intervene. Others were more deterrence-oriented, emphasizing fear of punishment, imprisonment, shame, or personal loss. This variation suggests that the court hearing did not produce a single or uniformly positive form of ethical intent. Rather, it prompted different forms of future-oriented moral reasoning, ranging from professional responsibility to consequence-based avoidance.

## 5 Discussion

From an educational psychology perspective, this study sheds light on how ethical sensitivity may be activated and articulated through students’ reflective accounts following an emotionally and institutionally salient experience. The findings indicate that attending a court hearing supported moral learning by activating a set of interconnected psychological processes involving affective engagement, reflective interpretation, and future-oriented reasoning. Rather than emerging from abstract ethical instruction, ethical sensitivity was triggered through students’ direct exposure to a real-world legal context in which moral responsibility was publicly examined but not yet resolved. This temporal and institutional positioning appeared to heighten learners’ attention to moral issues, disrupt habitual rule-based judgments, and create conditions conducive to deeper reflective processing. In this sense, the court hearing functioned as a psychologically meaningful learning environment that supported ethical sensitivity through dynamic affective–cognitive interaction, rather than simply as a source of moral content.

This study contributes to educational psychology research on moral learning in three interrelated ways. First, it conceptualizes court hearings as a temporally distinctive learning context in which moral responsibility is examined but not yet resolved, highlighting how real-time institutional processes shape learners’ psychological engagement. Unlike retrospective settings such as prison visits, court hearings expose learners to ethical judgment as it unfolds, allowing moral ambiguity, emotional tension, and institutional authority to coexist within the learning experience. Second, the findings identify empathic perspective-taking as a key psychological mechanism through which ethical sensitivity is activated in such contexts. Rather than treating empathy as an outcome in itself, the study demonstrates how imaginative self-placement supports reflective moral judgment by linking affective engagement with role-based responsibility. Third, the study extends research on experiential learning by showing how authentic legal processes can simultaneously support affective moral awareness, contextualized ethical judgment, and anticipatory ethical intent within a single learning episode. Together, these contributions advance understanding of how temporally situated institutional experiences support learning-related moral development beyond classroom-based or simulated interventions.

Research on moral learning has consistently suggested that ethical sensitivity is more likely to develop through engagement with psychologically salient experiences than through abstract instruction alone (Dewey, 1938; McPhail, 2003). Such experiences draw attention to moral consequences, evoke emotional responses, and prompt reflection on responsibility. This study contributes to this literature by showing how attendance at a court hearing shaped accounting students’ ethical sensitivity through interconnected affective, cognitive, and behavioral processes.

Consistent with Rest’s (1986) four-component model, students’ reflections indicate that ethical sensitivity was initially activated through their engagement with the court hearing. Emotional reactions to the courtroom setting, the defendant’s circumstances, and the public examination of misconduct heightened students’ awareness of the moral dimensions of professional wrongdoing. Rather than arising from abstract reasoning, ethical awareness was triggered through affective responses such as discomfort, empathy, and moral tension. This pattern reinforces prior research showing that emotional engagement plays a central role in directing attention to ethical issues in professional contexts, where moral considerations are often overshadowed by routine and technical concerns (McPhail, 2003).

A central contribution of this study lies in identifying empathic perspective-taking as a key psychological mechanism through which ethical sensitivity was supported and deepened following its initial activation. While previous research has emphasized the importance of empathy and perspective-taking in moral learning (McPhail, 2001; Bérubé and Gendron, 2023), the present findings demonstrate how imaginative self-placement enabled students to engage in more reflective and self-referential moral evaluation. Through this process, ethical judgment became more contextualized and relational, rather than detached or purely rule-based.

Importantly, empathic engagement did not result in moral leniency. Instead, students articulated more balanced judgments that distinguished between understanding situational pressure and excusing unethical conduct. Many were able to acknowledge personal hardship while affirming the necessity of professional and legal accountability. This capacity to hold compassion and responsibility together reflects a more advanced form of ethical judgment and aligns with educational psychology research emphasizing reflective evaluation over categorical moral reasoning (Dellaportas et al., 2022).

The court hearing also prompted students to reconsider how moral responsibility operates within institutional and professional roles. By observing how professional authority and specialized knowledge shaped the defendant’s capacity to intervene, students began to reassess assumptions about agency and obligation. Professionals were increasingly viewed not as constrained actors, but as moral agents with heightened responsibility. This finding supports educational psychology perspectives suggesting that ethical judgment is shaped not only by personal values, but also by learners’ reflection on role-based power, responsibility, and institutional context (Bérubé and Gendron, 2023).

Beyond moral awareness and judgment, students’ reflections also indicate the emergence of ethical intent as a forward-looking orientation. In line with Rest’s (1986) model, ethical intent was expressed through anticipated responses to future dilemmas. Rather than articulating abstract commitments to ethical conduct, students described concrete strategies such as refusing misconduct, seeking guidance, and drawing on organizational or personal support, suggesting a shift toward more agentic and proactive ethical reasoning.

The findings also suggest that ethical intent should be interpreted with caution. Some students articulated responsibility-oriented intent grounded in professional duty, whereas others expressed deterrence-oriented intent based primarily on fear of legal or personal consequences. This distinction indicates that future-oriented responses following the court hearing reflected varied forms of moral reasoning rather than a uniform professional ethical commitment.

Many students further described the court hearing as a lasting psychological reference point. The legal consequences and personal costs observed during the hearing were frequently cited as considerations they expected to recall when facing future ethical challenges. This suggests that ethical intent was shaped through moral imagination grounded in observed institutional outcomes, rather than through abstract deterrence alone. Similar effects have been noted in prior studies of experiential ethics education, particularly those examining prison visits that expose learners to the consequences of professional misconduct (Dellaportas and Hassall, 2013).

Taken together, these findings suggest that court hearings may provide a meaningful context for activating ethical sensitivity, supporting reflective ethical judgment, and encouraging students to articulate future-oriented ethical intent within a single experiential experience. Rather than prescribing specific moral conclusions, the experience invited students to engage with moral complexity, anticipate ethical challenges, and reflect on responsibility under realistic institutional constraints.

## 6 Conclusion

This study examined how engagement with an authentic legal setting may activate ethical sensitivity and prompt reflective moral sense-making within professional education. Drawing on accounting students’ reflective accounts, the findings indicate that attending a court hearing constituted a psychologically and institutionally salient learning experience that brought ethical awareness, moral judgment, and ethical intent into focus. By situating learners within a formal institutional process in which moral responsibility was examined publicly and in real time, the court hearing enabled students to engage with ethical issues as lived and consequential rather than abstract or hypothetical.

The findings suggest that ethical sensitivity was activated through emotionally and institutionally grounded experience. Students’ initial moral awareness emerged through affective responses to the courtroom environment and the defendant’s circumstances, which disrupted routine, rule-based evaluations. These emotional reactions did not determine moral conclusions but created conditions conducive to reflective engagement with ethical complexity. In this sense, the court hearing functioned as a psychologically meaningful learning catalyst that directed learners’ attention to moral issues and supported deeper reflective processing, consistent with the initial stages described in established models of moral decision-making.

Beyond moral awareness, the study highlights how ethical judgment developed through students’ engagement with competing values, particularly compassion and accountability. Students demonstrated an ability to acknowledge situational pressure while maintaining professional and legal responsibility. Rather than producing moral relativism, empathic engagement supported more proportionate and context-sensitive judgment. From an educational psychology perspective, this capacity to balance affective understanding with role-based responsibility reflects an important developmental outcome associated with reflective moral learning and is difficult to achieve through classroom-based instruction alone.

The findings further show that ethical intent emerged as a forward-looking orientation shaped by anticipation of real-world consequences. Students articulated concrete strategies for responding to future ethical dilemmas, including resisting misconduct, seeking guidance, and drawing on organizational or personal support. Importantly, the court hearing itself became a salient psychological reference point that students expected to recall when facing ethical pressure. Ethical intent was thus grounded in moral imagination informed by observed institutional outcomes, rather than in abstract moral aspiration.

By examining court hearings as a form of experiential learning intervention, this study extends existing research on field-based ethics education, which has largely focused on retrospective contexts such as prison visits (Castleberry, 2007; Dellaportas and Hassall, 2013) or on designed simulations (Van Akkeren and Tarr, 2021). The findings suggest that court hearings offer a distinct educational contribution by exposing learners to ethical judgment as it unfolds within authoritative institutional processes. This real-time engagement appears particularly well suited to supporting reflective ethical judgment and more durable ethical intent through psychologically salient learning experiences.

Several limitations should be acknowledged. First, the study was conducted in a single institution and focused on one cohort of undergraduate accounting students. Although the participants provided rich reflective narratives, the findings may not be directly transferable to other institutions, disciplines, or student groups. In addition, because students first expressed interest in attending the court hearing, the final sample may have included students with a pre-existing interest in ethical, legal, or professional responsibility issues. Second, the study did not include baseline or follow-up qualitative data. As a result, the analysis cannot determine how students’ reflections differed from their prior views, nor whether the responses observed immediately after the court hearing persisted over time.

Third, the study relied on post-event self-reported reflections. These data are appropriate for examining students’ articulated interpretations, moral sense-making, and perceived future intentions, but they do not provide evidence of actual behavioral change. The study therefore cannot determine whether students will engage in, or avoid, unethical conduct in future professional settings. Students’ stated intentions to resist misconduct, seek guidance, or act ethically in future situations should therefore be interpreted as reflective and prospective accounts rather than observed professional behavior.

Finally, the cultural and legal context of the study should be recognized. The court hearing took place within a specific Chinese legal and educational context, and students’ responses may have been shaped by local understandings of legal authority, professional responsibility, family obligation, and public accountability. The meaning and educational impact of court hearing engagement may therefore differ across jurisdictions, legal systems, and educational traditions. Future research could examine similar court-based or legal-institutional learning experiences across different cultural and disciplinary contexts, incorporate baseline and delayed follow-up data, and use complementary methods to explore whether students’ reflective responses persist and translate into professional action.

Overall, this study suggests that court hearings can function as meaningful sites for reflective moral learning within professional education by bringing together affective engagement, reflective judgment, and future-oriented ethical intent within a single experiential context. From an educational psychology perspective, such experiences support learners’ capacity to navigate moral complexity under realistic institutional constraints, highlighting the value of incorporating authentic legal contexts into educational designs aimed at fostering ethical sensitivity as a developmental learning outcome.

## Data Availability

Due to ethical considerations, confidentiality requirements, and the potential risk of participant identification, the raw data cannot be shared publicly or made available upon request. Requests to access the datasets should be directed to the corresponding author.

## Appendix

**Table A1:** Summary of participant codes by theme and sub-theme.

Main theme	Sub-theme	Participant codes	Illustrative example
Affective moral awareness	Initial negative impression	0539	“My first impression of her was not good…”
Affective moral awareness	Sympathy and emotional tension	0527, 0806, 0534	“I sense that Anna was compelled by her circumstances…”
Reflective ethical judgment	Professional responsibility	0622	“If Anna had refused from the outset…”
Future-oriented ethical intent	Responsibility-oriented intent	1129, 0952	“I will try to dissuade the individual…”
Future-oriented ethical intent	Deterrence-oriented intent	1087, 0536	“The thought of facing the same fate as Anna…”
